# Assessment of Regional Health Resource Carrying Capacity and Security in Public Health Emergencies Based on the COVID-19 Outbreak

**DOI:** 10.3390/ijerph20032068

**Published:** 2023-01-23

**Authors:** Xiaoran Huang, Demiao Yu

**Affiliations:** 1 School of Architecture and Art, North China University of Technology, Beijing 100144, China; 2 Centre for Design Innovation, Swinburne University of Technology, Hawthorn, VIC 3122, Australia

**Keywords:** health resources, COVID-19, public health emergencies, risk assessment

## Abstract

The Omicron variant of COVID-19, which emerged at the end of 2021, has caused a new wave of infections around the world and is causing a new wave of the crisis due to the extreme variability of the pathogen. In response to public health emergencies such as SARS and COVID-19, the first task is to identify the vulnerabilities of regional health systems and perform a comprehensive assessment of the region’s resilience. In this paper, we take the carrying capacity of medical resources as the focus; evaluate the medical, human, and financial resources of various regions; and construct an epidemic safety index based on the actual situation or future trend of the epidemic outbreak to evaluate and predict the risk level of each region in response to the epidemic. The study firstly evaluates the epidemic safety index for each province and city in China and 150 countries around the world, using the first wave of the COVID-19 epidemic in 2020 and the Omicron variant virus in 2022 as the background, respectively, and justifies the index through the actual performance in terms of epidemic prevention and control, based on which the epidemic safety index for 150 countries in the next year is predicted. The conclusions show that Europe, the Americas, and parts of Asia will face a significant risk of epidemic shocks in the coming period and that countries need to formulate policies in response to the actual situation of the epidemic.

## 1. Introduction

### 1.1. Background

Since the World Health Organization declared an outbreak of COVID-19 [1], the virus has been raging worldwide for two and a half years. According to the latest WHO report, as of 31 August 2022, there were more than 600 million confirmed cases of COVID-19 in more than 188 countries and territories, including 6.46 million deaths [2]. COVID-19 has become the most serious public health event in the world today [3].

Since the outbreak of COVID-19, SARS-CoV-2 as the causative agent behind it has shown extreme variability. So far, it has produced five significant variants, namely Alpha (B.1.1.7), Beta (B.1.315), Gamma (P.1), Delta (B.1.617.2), and Omicron (B.1.1.529). Studies have shown that the Omicron (a variant of SARS-CoV-2) strain that emerged in late 2021 is significantly more infectious than the Delta strain that was prevalent in early 2021 [4]. To date, there are over 100 subvariants of Omicron, and new subvariants are constantly being created [5].

In public health emergencies such as the COVID-19 epidemic, there are often several concerns: firstly, overall resources are given and cannot be increased rapidly in the short term, so increases in one location are accompanied by decreases elsewhere [6]; secondly, virus transmission is highly unpredictable [5], and there are still unknowable weaknesses in the long battle lines of epidemic preparedness.

Therefore, with the huge wave of infection with the Omicron variant, there is an urgent need to assess the level of risk in each country and region of the world in the face of the new phase of the epidemic, in order to guide the deployment of medical supplies and the development of epidemic prevention policies in each country and region.

### 1.2. Literature Review

Major epidemics differ from natural disasters: the former are highly contagious, while the latter rarely spread elsewhere [7]; the former are highly demanding of trained healthcare professionals and specific medical equipment [6], while the latter have a broader spectrum of resource requirements [8]; the secondary hazards of the former are hidden but frequent, while those of the latter are relatively visible and controllable. As a result, major epidemics create tremendous economic, social, and international relationship pressures and demands for health resource mobilization and deployment [9]. Therefore, regional healthcare resources are essential in evaluating a city’s risk level under the impact of the COVID-19 outbreak.

Where resources cannot be increased in the short term, regional differences in healthcare resources will constitute a structural constraint on resilience [9]. Currently, the healthcare resources are highly unevenly distributed across countries globally and even within countries by region [10,11]. The uneven distribution of healthcare resources within countries and between countries in regions such as Cambodia [10], China [12], Canada, Australia [13], and the Greater Mekong Subregion [11] has been demonstrated in studies addressing healthcare resources.

Although regional differences in healthcare resources interfere to some extent with epidemic prevention, the defensive capacity of this system does not depend entirely on the absolute number of hospitals and amount of equipment at the top end of each country and region, but equally on the sustainable supply of these resources and on whether weaknesses in healthcare resources are identified and compensated for, given the indiscriminate attack launched by the virus.

Similarly, the judgement of the risk level for an outbreak area and the method and efficiency of government emergency management equally affect the effectiveness of the outbreak prevention and control initiatives [14]. Thus, following the COVID-19 outbreak, there has been a proliferation of research in the academic community on emergency management and risk assessment for the COVID-19 outbreak [15]. 

In emergency management studies, COVID-19 outbreak prevention and control approaches for travel [16], transport [17], and community [18] have achieved significant management performance. At the same time, research has been conducted on a range of social issues arising from the COVID-19 epidemic, such as the panic buying behavior of the public [19], the differential prevention strategies of people in different regions due to their cultural orientation [20], and the relationship between urban prosperity and the spread of the COVID-19 epidemic [21]. 

Most of the studies on the risk assessment of public health emergencies are based on people who live in cities, including simulations of people’s behavior [22], the determination of population movement patterns based on mobile phone data [23], and assessments of the public’s ability to cope with risk during public health emergencies from a psychological perspective [24]. At the same time, the application of new technologies has also played an essential role in risk assessment; establishing the Smart City Risk Assessment System will provide technical support for accurate risk assessments in cities [25]. As for the layers of risk assessment, there is a rapid risk assessment system established specifically for cases imported from abroad at ports [26], a two-level risk assessment method for cities with a macro–micro combination [27], and an assessment method for risk at the city cluster level based on the spread characteristics of the COVID-19 epidemic [28].

However, the current research on the management and assessment of epidemic risk has tended to focus on a particular part of urban management for the development of epidemic prevention and control strategies or the determination of the risk level of a city based on a single factor, such as the transmission characteristics of the virus or the behavior of the population. In general, there is a lack of systematic and complete risk assessments of epidemic areas based on the vital factor of medical resources. 

### 1.3. Framework

In summary, relying on the vital factor of medical resources, this paper aims to establish a risk evaluation system for responding to public health emergencies. The system comprehensively assesses the risk of outbreaks in different regions by combining medical resources and the actual outbreak situation in each region. It can identify the high-risk areas of epidemic outbreaks and the weak links in epidemic prevention and control, providing a reliable basis for deploying medical resources and formulating epidemic prevention and emergency management policies.

To this end, we developed a “health resource carrying capacity” model and an “infection rate during the period” model to fairly evaluate the health resources available in each region based on the difference in population size and to evaluate (or predict) the severity of epidemic infection in each region. Based on these two models, they are integrated to build a “COVID-19 safety index” indicator system, which not only presents a more balanced and objective picture of the possible vulnerabilities in epidemic prevention and control work and the focus of deployment but also helps to promote the equalization of regional healthcare resources and fundamentally improve the risk resistance of the healthcare system.

**Figure 1:**
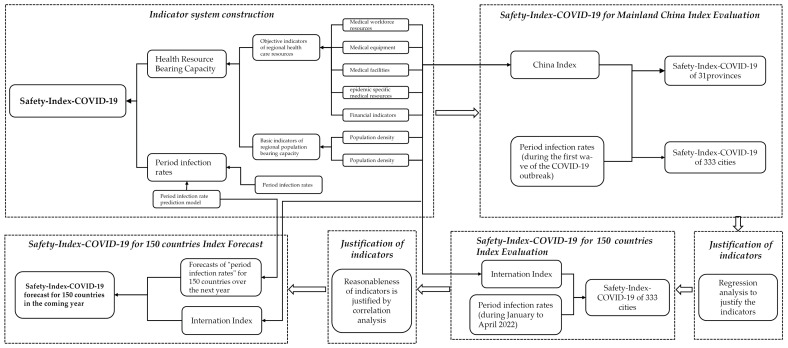
Research framework.

Due to the suddenness and uncertainty of public health emergencies, preventing and controlling the first wave of the COVID-19 outbreak can reflect the actual carrying capacity and accurate risk level of health resources in each region when there is no preparation. Therefore, this study constructs a COVID-19 safety index model for 31 provinces and 333 prefecture-level cities (excluding areas without outbreak infection) in mainland China [29], where the first wave of COVID-19 epidemic prevention and control was most effective in 2020, confirming its validity. Based on this, we construct a COVID-19 safety index for 150 countries around the world (excluding countries with incomplete data) based on the epidemic data from January to April 2022 and further justify its rationality by using the data from April to August. Finally, we use a machine learning prediction model to predict the epidemic situation in each country in the next year and evaluate the epidemic safety index of each country in the next year in order to provide a basis and reference for the formulation of epidemic prevention and control policies and the allocation of medical resources in the context of the rampant Omicron variant around the world.The specific framework is shown in Figure 1.

The article is organized as follows: After a short introduction, we discuss in Section 2 how the concepts and models of the “health resource carrying capacity”, “period infection rates“, and “COVID-19 safety index” were constructed, as well as the basis for the selection of all variables and data sources. Section 3 describes the results of the evaluation of the resource carrying capacity and the COVID-19 safety index for mainland China. The fourth section evaluates and predicts the COVID-19 safety index values for 150 countries worldwide. The fifth section addresses the analysis of the results and reflections on the study’s limitations. The sixth part is a long-term reform proposal for the vulnerabilities revealed by the model. 

## 2. Materials and Methods

### 2.1. Construction of the “Health Resource Carrying Capacity” Model

#### 2.1.1. The Indicator System of the “Health Resource Carrying Capacity” Model

An objective assessment of the inter-regional resource carrying capacity is this paper’s primary objective and basis. Currently, there are significant differences between countries and regions in terms of the population structure and distribution, the economic development and financial capacity [30], and the quantity and allocation of health resources [31,32]. These regional differences in the distribution of financial, demographic, and health resources profoundly affect the inputs, performance, and health outcomes of a region’s health system in the face of a public health emergency [33]. Therefore, this paper evaluates three critical resources, including the population, economics, and healthcare, and constructs a three-level evaluation index system for the “healthcare resource carrying capacity” model (to evaluate the carrying ability of a region to cope with the impacts of a public health emergency with its current population, financial, and medical resources) hereafter called the HRCC model. 

The assessment of the carrying capacity of a region’s healthcare resources to cope with public health emergencies depends, on the other hand, on the specific situation of the object that carries them—the population. Therefore, in constructing the first indicators, they are divided into “objective indicators of regional healthcare resources “and “basic indicators of the carrying capacity of the region’s population”.

In the construction of objective indicators for regional healthcare resources, the paper divides them into five secondary indicators, such as medical workforce resources, resources for medical equipment and facilities, unique medical resources for epidemics, and corresponding financial resources, which largely determine the ability of a region to respond to an outbreak or other public health event. Firstly, medical workforce resources have a significant impact on health outcomes in the health system [34,35]; play an essential role in identifying, treating, and managing infected people with infectious diseases and in limiting the spread of infection; and are even seen as central to the functioning of the healthcare system [36]. Moreover, unlike financial and material resources, medical workforce resources are more clearly “stock” and cannot be increased in the short term. Therefore, doctors, nurses, medical technicians, and CDC staff are used as tertiary indicators of what constitutes human resources for healthcare (international indicators were not included in the CDC staff due to data availability issues). Secondly, focusing on the COVID-19 outbreak, the diagnosis and treatment of this type of severe acute respiratory syndrome rely heavily on medical equipment such as CT machines, ventilators, and ambulances [37], which will, therefore, be considered a tertiary evaluation indicator for medical equipment resources. Thirdly, the numbers of ICUs and beds, as common indicators for measuring medical facilities or physical resources, constitute one of the necessary foundations for effective healthcare system functioning [38], constituting a tertiary indicator for the resources of medical facilities. It is important to note that the construction of international indicators is limited by the inconsistency of the statistical caliber of those medical resources in each country and the different situations of disclosure, so we adopted the HAQ (Healthcare Access and Quality) indicator [39] as a tertiary indicator to measure medical equipment and facilities in other countries internationally. Last but not least, unique medical resources during an epidemic are often allocated in a targeted manner according to the actual situation of the epidemic. Therefore, different indicators need to be adopted for evaluation during different phases of the epidemic. In the construction of indicators for the COVID-19 outbreak in China in 2020, facilities such as the “COVID-19 case intensive care hospitals”, “fever clinics”, and “hospitals able to treat COVID-19 patients” [40] were used as the tertiary indicators; in the evaluation of specific medical resources for the 2022 Omicron variant outbreak in international countries, the number of COVID-19 vaccinations [41] and the “average score of 13 IHR core competencies” from the World Health Statistics 2022 report [42] were used as the corresponding tertiary indicators.

The final component of the index construction is the regional financial expenditure variable, and the large consumption of health resources is dependent on local solid financial support. The resilience of the healthcare system is not only a function of the healthcare system alone but also of the economic and social system as a whole [33,43,44]. Therefore, we did not limit ourselves to fiscal expenditures in healthcare alone but used government revenues and expenditures as a tertiary indicator for measuring economic resources.

The construction method of the basic indicators of the regional population carrying capacity takes the population distribution and population structure as he secondary evaluation indicators. The population density in an area influences to a certain extent the extent to which public health emergencies such as the SARS and COVID-19 epidemics spread in an area, and often less densely populated areas are affected by the epidemic to a lesser extent [45]. In the current study of the COVID-19 epidemic, it was found that people of different ages were not affected by the epidemic to the same extent, and often older people were exposed to greater risk of the epidemic [46]. Therefore, the population density and degree of ageing are tertiary indicators used to measure the population distribution and demographic structure.

In summary, the composition of the three-level evaluation indicators for the carrying capacity of healthcare resources in this paper is shown in Table 1 (to better reflect the carrying capacity of health resources, we consider both the absolute total and the distribution density of each indicator).

#### 2.1.2. Determination of Indicator Weights (Entropy-Weighted TOPSIS Method)

(1) Justification for weighting options

Currently, the methods used to determine the weights in the construction of indicator systems can be broadly divided into three categories: subjective assignment methods, objective assignment methods, and subjective–objective assignment methods. Among them, the subjective weighting methods are mainly the analytic hierarchy process (AHP) [47] and Delphi method [48], which are widely used. The prevailing objective assignment methods are the principal component analysis (PCA) [49], entropy-weighted [50], and factor analysis methods [51]. The objective and subjective weighting methods are most widely used by the linear weighted sum method [52], the catastrophe progression method (CPM) [53], and the Grey relational analysis (GRA) [54].

In this study, the entropy weighting method was chosen to determine the weights of the HRCC indicators for the following reasons. Firstly, the AHP and Delphi methods are subjective assignment methods, which are widely used but less objective [55]. In the case of the HRCC indicator system in response to public health emergencies, the distribution of various anti-epidemic materials will change over time, so a purely subjective assignment method will introduce arbitrary and unobjective bias to the results. Secondly, the factor analysis inevitably results in a loss of information when the principal components are extracted quickly, and the loss of information is positively related to the number of indicators [56]. Thirdly, the CPM draws on the advantages of the hierarchical analysis and fuzzy evaluation methods, relying on the potential function to assign scores to indicators. However, due to the limited type of potential function, the number of evaluation indicators is generally less than four [57], which greatly limits the number of evaluation factors in the HRCC evaluation system and does not have the advantage of comprehensiveness. Fourthly, the GRA is mainly used for the later classification of the hierarchical analysis or factor analysis in the comprehensive evaluation, and their independence is poor [58]. Various medical resources in the HRCC indicator system in response to public health emergencies will produce changes in the type and quantity of indicators with the stage changes of the epidemic. They cannot completely avoid the influence of subjective human factors. Thus, they are not suitable as methods for assigning weights to HRCC indicators.

In this study, the entropy-weighted TOPSIS, AHP, factor analysis, and GRA methods were used to assign weights to the indicators for each city in China to test each method’s feasibility further. The Pearson correlation coefficients correlated each outcome with the epidemic prevention and control performance separately. The results are shown in Appendix A.

From the correlation analysis, it can be seen that the COVID-19 safety index results obtained from the weights assigned by the entropy-weighted TOPSIS method have the most significant correlation results with the performance of the resistance to the epidemic in the COVID-19 outbreak.

In conclusion, this paper adopts the entropy-weighted TOPSIS method as the way to assign weights to HRCC indicators.

(2) Calculation of the Entropy-Weighted TOPSIS Method

The entropy-weighted TOPSIS method is essentially an improvement of the traditional TOPSIS evaluation method, in which the weights of the evaluation indicators are determined by the entropy method. Then, the ranking of the evaluation objects is determined by the TOPSIS method using the technique of approximating the ideal solution [50]. The entropy weighting method is based on the information provided by each evaluation indicator and objectively determines the weight as the entropy weight. It not only objectively reflects the importance of an indicator in the indicator system at the time of decision-making but also prominently reflects the changing status of the weight of the indicator over time [59]. Therefore, it is very suitable for the evaluation study of the HRCC values in different periods. Moreover, the core idea of the TOPSIS method is to determine the distances between the optimal and inferior solutions of the decision problem, calculate the relative fit of each solution to the ideal solution, and rank the superiority of each solution [60]. The specific implementation steps are as follows:

Step 1: Data normalization

For positive-type indicators:

(1) $$Y_{ij}=\frac{X_{ij}-min\left(X_{j}\right)}{max\left(X_{j}\right)-min\left(X_{j}\right)}$$

For inverse-type indicators:

(2) $$Y_{ij}=\frac{max\left(X_{j}\right)-X_{ij}}{max\left(X_{j}\right)-min\left(X_{j}\right)}$$

Here, $X_{ij}$ is the original indicator data, $max\left(X_{j}\right)$ is the maximum value of the *j*th indicator data, $min\left(X_{j}\right)$ is the minimum value of the *j*th indicator data, and $Y_{ij}$ is the normalized data value.

Step 2: Calculate the entropy value for each indicator

When calculating the entropy value, to avoid incorrect values, the standardized data are shifted to the right by 0.0001 units:

(3) $$E_{j}=-\frac{1}{ln\left(n\right)}\sum_{i=1}^{n}p_{ij}lnp_{ij}$$

Here, the *p_ij_* value is calculated as follows:

(4) $$p_{ij}=\frac{Y_{ij}}{\sum_{i=1}^{n}Y_{ij}}$$

Here, *n* is the number of healthcare resource carrying capacity indicators and *E*_*j* is the entropy value of the indicators.

Step 3: Find the variability coefficient of each indicator:

(5) $$g_{j}=1-E_{j}$$

Step 4: Find the weight of each indicator:

(6) $$W_{j}=\frac{g_{j}}{\sum_{j=1}^{m}g_{j}}$$

Here, *m* is the number of indicators and $W_{j}$ is the *j*th indicator weight.

Step 5: Derive the weighted standardized data:

(7) $$Z_{ij}=W_{j}\times Y_{ij}$$

Here, $Z_{ij}$ represents the weighted standardized data.

Step 6: Determine the positive and negative ideal solutions:

(8) $$Z_{j}^{+}=\left\{max\left(Z_{ij}\right)|i=1,2,\cdots ,n\right\}$$

(9) $$Z_{j}^{-}=\left\{min\left(Z_{ij}\right)|i=1,\;2,\cdots ,n\right\}$$

Step 7: Find the Euclidean distance of each evaluation object to the positive and negative ideal solutions in turn:

(10) $$D_{i}^{+}=\sqrt{\overset{m}{\underset{j=1}{\sum }}\left(Z_{ij}-Z^{+}\right){\;}^{2}}$$

(11) $$D_{i}^{-}=\sqrt{\overset{m}{\underset{j=1}{\sum }}{\left(Z_{ij}-Z^{-}\right)}^{2}}$$

Here, $D_{i}^{+}$ is the Euclidean distance from the *i*-th evaluation object to the positive ideal solution; $D_{i}^{-}$ is the Euclidean distance from the *i*-th evaluation object to the positive ideal solution.

Step 8: Calculate the relative closeness:

(12) $$C_{i}=\frac{D_{i}^{-}}{D_{i}^{+}+D_{i}^{-}}$$

Here, *C_i_* represents the composite development index of the *i*th healthcare resource carrying capacity evaluation index.

After calculating the entropy-weighted TOPSIS Method, the final weight distribution is as follows: limited by length, only the weighting scores of 31 provinces in mainland China (Table 2) and 150 countries worldwide (Table 3) are shown here.

### 2.2. Prediction-Period Infection Rate Model

#### 2.2.1. Period Infection Rates

(1) Measurements of outbreak transmission rates

After assessing the resource carrying capacity of a region, it is necessary to measure and compare the impacts of regional differences in epidemic risk on the demographic characteristics of the region. Therefore, this paper combines public health research on infectious disease infection rates and research needs to construct this paper’s epidemic risk index—the period infection rate. Various methods of measuring disease, including the attack rate, incidence rate, and prevalence rate, are often used for different data [61]. The prevalence rate, which is more commonly used in the measurement of infectious diseases, refers to the ratio of the number of cases of a disease (new and old) in a given population to the average population over the same period [62]. The prevalence rates can be divided into period prevalence rates and point prevalence rates.

(2) Construction of the period infection rate

The prevalence rates provide a more objective measure of the severity of infectious disease at a particular time or period in a region. However, this paper uses them in a modified way for the following reasons. Although they measure the severity of an epidemic in a region, prevalence rates ignore intra-group differences in groups of infected persons with an epidemic, such as the simple classification of those with severe, moderate, and mild infections, or those with common infections and those with classifications such as “super-spreaders” would have significantly different implications for issues such as regional outbreak medical resources and the evolution of the epidemic. To avoid similar issues having a misleading effect on the risk assessment of the epidemic, we adapted the calculation of the period prevalence rate to the sum of the cumulative number of infections during the epidemic, divided by the total annual population of the region, to obtain the “period infection rate”, which is calculated as follows:

(13) $$Ir_{i}=\frac{\sum_{t=1}^{n}I\left(t\right)}{N}$$

*Ir_i_* indicates the periodic infection rate (the ratio of the total number of infections during the cumulative period of the epidemic to the total annual population of the area), where $\sum_{t=1}^{n}I\left(t\right)$ indicates the total number of infections during the cumulative period of the epidemic (where *n* indicates the actual number of days of the epidemic in an area) and *N_i_* indicates the total population of area *i*.

The “period infection rate” is corrected to calculate the total number of infections during the epidemic because the consumption of regional medical resources does not depend on the specific citizen that is infected but on the number of infected persons per day, and it is only when an individual changes from “infected” to “cured” that the consumption of medical resources ends. Therefore, a more accurate measure of the regional epidemic risk index is based on a combination of the number of people infected and the number of days of epidemic conditions.

#### 2.2.2. COVID-19 Epidemic Risk Prediction Model: LTSM Prediction Model

(1) Reasons for model selection

The LSTM model is a temporal recurrent neural network model. Due to its unique design structure, LSTM is suitable for processing and predicting time series tasks with long intervals and delays in the time series. Long before the COVID-19 outbreak, LSTM models were used to predict the trend of respiratory disease transmission [63]. After the COVID-19 outbreak, LSTM models were widely used to predict the COVID-19 outbreak and showed good results. For example, Yang et al. [64] used the SARS outbreak data in 2003 to train the LSTM model and then used the trained model to predict the epidemic trend of the new crown pneumonia outbreak. Pal et al. [65] proposed an LSTM model with an improved parameter training method to predict the infection rate of the new crown outbreak and determine the risk level of different countries based on the prediction results. Rauf et al. [66] compared the prediction effectiveness of RNN, LSTM, and GRU networks on epidemic datasets from countries in the Asia-Pacific region, such as Pakistan and Afghanistan. Therefore, we used the LSTM model to predict the number of infections in the COVID-19 epidemic.

(2) LSTM prediction model

**Figure 2:**
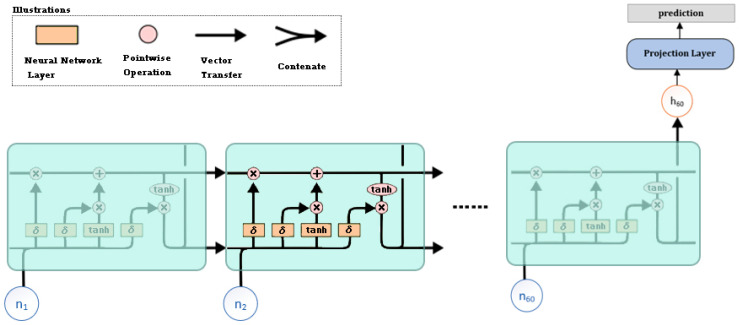
Illustration of LSTM model structure.

The basic idea of our model is to use the number of infections in the first 60 days to predict the number of infections in the next days, until all infections are predicted. Specifically, we first take the number of infections in the first 60 days as the input of long short-term memory (LSTM) [67] units. Then, the hidden state of the last time step in the LSTM, followed by a projection layer, which is a single feed-forward neural network, is used to predict the infections the next day. All experiments are conducted on a single NVIDIA 1080Ti GPU. The hidden size of the LSTM unit is set to 512. During training, we optimize the model’s parameters using the Adam optimizer [68], using the learning rate of 5 × 10^−4^, and the objective function is the mean-square error (MSE) loss. The model structure is shown in Figure 2.

### 2.3. COVID-19 Safety Index

Examining the adequacy of the resilience of various parts of the healthcare system or regions requires a combination of real-time updates on the current state of risk resilience and the likely sources and magnitude of future risks [37], so this paper further constructs the “COVID-19 safety index” to measure the resilience of the health system to risk. The “COVID-19 safety index” reflects how many units of resources are available on average for specific risk prevention and control to combat risk, meaning it requires the combination of the regional resource carrying capacity and risk level. Based on the previous research, we combine the HRCC with the “period infection rate” as a ratio. Unlike the single infectious disease models currently used by countries and regions, the “COVID-19 safety index” provides a more comprehensive and balanced picture of regional epidemic risk.

### 2.4. A Note on the Selection of the Epidemic Phase

In this paper, the following considerations were made in selecting the development phase of the epidemic. Firstly, as China adopted dynamic clearance measures for the COVID-19 outbreak in early 2020, which mainly reflects the complete process of public health emergencies from their creation to their end, this period was chosen as the evaluation phase for China. Secondly, the outbreak from January to April 2022 was selected as the evaluation phase for the analysis of countries around the world. This was because the Omicron variant emerged in late 2021 and spread rapidly across the globe in early 2022, reflecting to some extent the state of a new and unexpected public health emergency when it first broke out.

After the experimentation and evaluation of a full round of outbreaks, as well as a period of outbreaks, the HRBC and COVID-19 safety index were proven effective in predicting, evaluating, and reflecting the level of risk in each region’s response to an outbreak, both in the early stages of an outbreak and after it had ended. The establishment of the HRBC and COVID-19 safety index indicators provides a complete and valid set of indicators and systems for measuring the overall strength of health resources and the level of risk in response to other upcoming public health outbreaks in the future. This is one the most significant contributions of this study.

Finally, the significance of choosing different periods and measurement objects is to argue the applicability of the evaluation system in different stages of epidemic development rather than comparing them. In addition, in calculating international indicators, the epidemic data in China were collected at the same time as other countries, from January to April 2022. All data for China’s healthcare resources were from the same sources as other countries. This ensured the comparability of the data.

## 3. COVID-19 Safety Index Evaluation (by Provinces and Cities in Mainland China) and Its Reasonableness Argument

Based on the 2020 population, medical, and financial data for 31 provinces and 322 prefecture-level cities in mainland China, we constructed a medical resource carrying capacity model to analyze the distribution of their medical, financial, and population resources and evaluate their epidemic safety indices in the first wave of COVID-19 in 2020. After evaluating the epidemic safety index, the rationality of the epidemic safety index was justified by conducting a one-dimensional linear regression analysis of its correlation indicators with the actual status of the recovery of the epidemic in each place.

### 3.1. Evaluation of HRCC by Provinces and Cities in Mainland China

#### 3.1.1. Analysis of HRCC Capacity Indicators

**Figure 3:**
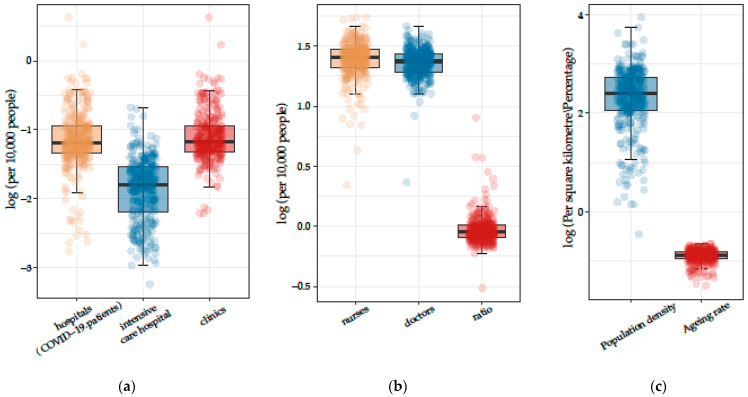
Distribution of HRCC key indicators by cities: (**a**) epidemic-specific medical resources; (**b**) medical workforce resources; (**c**) population indicators (data sources: Provincial Health Statistical Yearbook; Municipal Health Committee Bulletins; Municipal “National Economic and Social Development Statistical Bulletins”; China’s Seventh Population Census Bulletin).

Firstly, after nearly a decade of development, the regional distribution of healthcare resources between Chinese provinces remains uneven (Qin and Hsieh, 2014). In order to objectively compare the overall performance of the indicators between provinces and municipalities, we carried out a graphical analysis of the indicators (distribution density) of the HRCC for 31 provinces (Table 4) and 333 municipalities (Figure 3).

Table 4 shows the uneven distribution of healthcare resources and financial expenditure among the country’s provinces. Firstly, the province with the highest number of beds per capita also reached 1.8 times that of the smallest province. The interprovincial best value ratios for licensed physicians and registered nurses reached 2.98 times and 3.21 times greater, respectively. Secondly, the per capita fiscal expenditure best-value ratio was nearly six times greater, while the fiscal ratio for medical expenditure was only 1.77 times greater. Finally, the statistics on healthcare resources for specific epidemics show that the amounts of healthcare resources allocated to the COVID-19 epidemic vary greatly between regions, which may be related to the status of the local epidemic.

**Figure 4:**
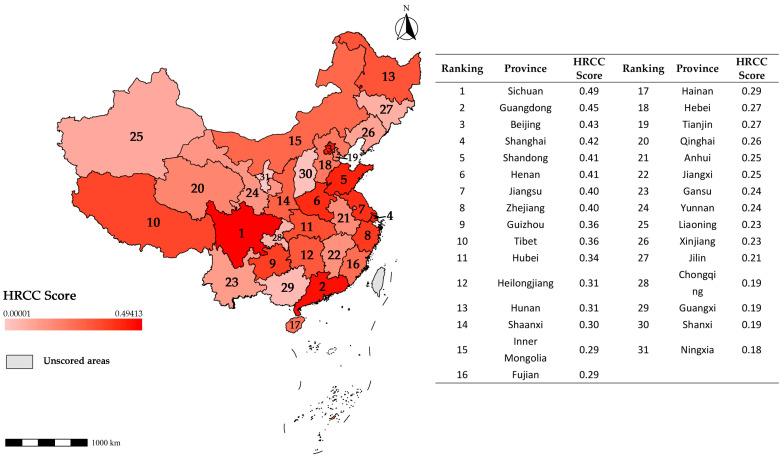
The HRCC scores in 31 provinces in mainland China.

After analyzing the provincial data, we selected important indicators such as the medical workforce and epidemic-specific medical and population resources in each city for a further detailed regional analysis (Figure 3). According to Figure 4, it can be seen that there are clear regional differences in the distribution of epidemic-specific medical resources in terms of general practice hospitals. The urban differences between doctors and nurses are close, but the figures for the ratio of doctors to nurses are not ideal. In the graphical representation of the population resources, it can be seen that the regional differences in population density are much higher than the ageing values for each region.

#### 3.1.2. Evaluation of HRCC in Provinces and Cities in Mainland China

**Figure 5:**
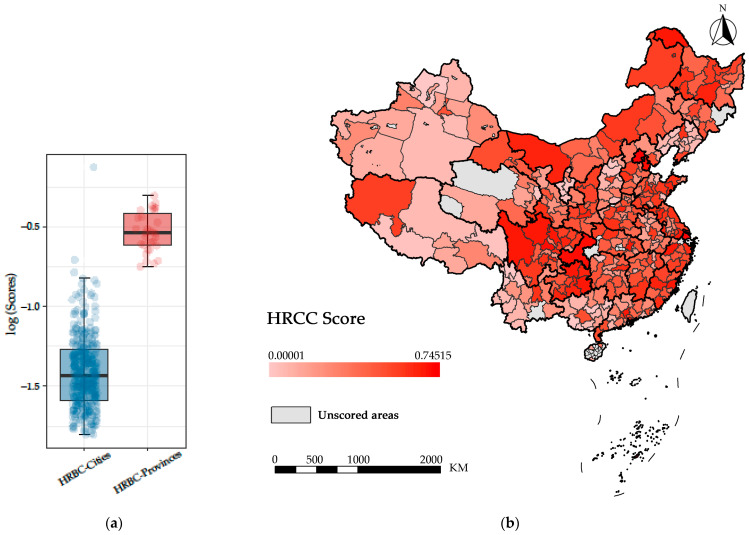
HRCC scores and comparisons: (**a**) provincial and municipal HRCC score statistics; (**b**) the HRCCs of 333 cities in mainland China.

In order to visualize the state of HRCC in each province and city in China, we constructed Figure 4 and Figure 5 based on the calculated HRCC scores. In Figure 4, the lighter the color, the higher the carrying capacity of the province. The coastal provinces generally have higher HRCC scores, while the northeastern and western regions generally have lower HRCC scores.

Figure 5 further demonstrates the HRCC scores of the cities and the differences between them and their provincial scores: firstly, while there are clear imbalances in the distribution of resources between provincial administrative units, the differences are more pronounced at the subprovincial level within provinces; secondly, some less economically developed cities in central and western China have higher scores, such as some cities in Tibet and Xinjiang, which may rely on their smaller populations and higher density of resource distribution to achieve a higher rating.

### 3.2. COVID-19 Safety Index Evaluation of Provinces and Cities across Mainland China for the 2020 COVID-19 Outbreak

**Figure 6:**
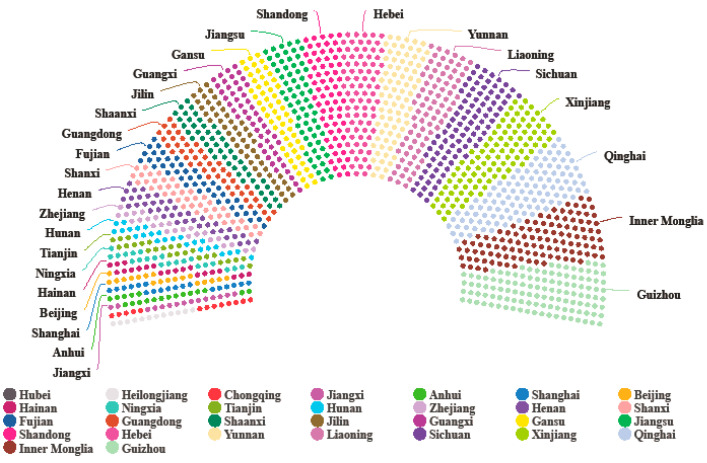
Distribution of the “COVID-19 safety index” scores in 333 cities in mainland China (an extreme value was excluded because there was only one case in Tibet, resulting in a high safety index).

**Figure 7:**
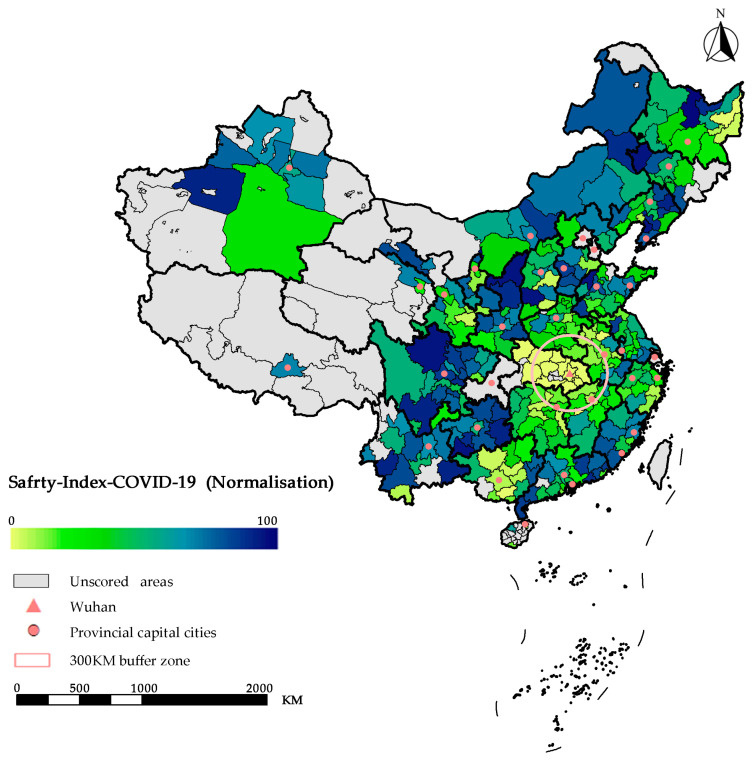
Distribution of the “COVID-19 safety index” scores in 333 cities in mainland China.

Based on the actual situation of the COVID-19 outbreak in 2020, this paper calculates the period infection rate of each province and city in mainland China, combines the HRCC scores of each place, evaluates the safety of the outbreak in each place, and draws Figure 6 and Figure 7 based on the COVID-19 safety index score of each province and city.

In particular, Figure 6 represents each province’s distribution of the COVID-19 safety index scores. The larger the area of the sector occupied, the higher the COVID-19 safety index score. Figure 7 reflects the level of safety in mainland Chinese cities in the case of a COVID-19 outbreak in 2020. In this case, darker colors indicate lower risk, and vice versa. Additionally, using Wuhan, the center of the outbreak, as a circle representing the radius of the outbreak area, it can be seen that most of the cities around Wuhan are affected by the outbreak and have a lower COVID-19 safety index scores.

Combining the distribution of the medical resource carrying capacity in Figure 4 and Figure 5, we find that the degree of regional risk depends to a certain extent on the regional medical resource carrying capacity. For example, most cities in the eastern coastal regions such as Shandong, Jiangsu, and Zhejiang have relatively low risk due to their relatively good medical resource carrying capacity, despite the scale of infection and the serious risk of the epidemic. However, at the same time, we cannot ignore the impact of the scale of the epidemic, such as in Tibet and Qinghai, where the safety index scores are relatively high due to the small scale of the actual infection.

### 3.3. The Reasoned Argument Analysis of the COVID-19 Safety Index

This paper uses least squares estimation (OLS) to analyze the validity of the COVID-19 safety index scores, specifically the correlation between the epidemic safety index and the actual epidemic control performance for 31 provincial-level regions and 320 prefectural and municipal-level regions in China, respectively, with the following estimation formulae (due to the limitation of space, this section only shows the correlation analysis for 320 prefectures):

(14) $$\mathrm{Sisuation}\_\mathrm{COV}19_{i}={\alpha \mathrm{Index}}_{i}+C+\mu_{i}\;(C\;\mathrm{is}\;a\;\mathrm{constant},\;\mu_{i}\;\mathrm{is}\;\mathrm{residual})$$

**Figure 8:**
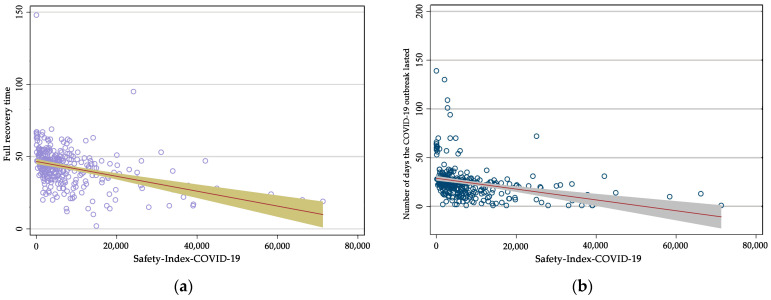
Correlation diagrams: (**a**) the correlation analysis of the COVID-19 safety index with the number of full recovery days; (**b**) the correlation analysis of the COVID-19 safety index with the number of days the outbreak lasted.

The results of the COVID-19 safety index analysis are presented in Figure 8 and Table 5, which show that the COVID-19 safety index correlates strongly with the number of days to peak infection, the number of days to recover from infection, and the number of days the epidemic lasts, but not with the number of cumulative infections, the number of peak infections, or the number of deaths. This shows that the COVID-19 safety index adequately reflects the efficiency of the fight against the epidemic in each region and its reasonableness is justified.

## 4. Evaluation of the COVID-19 Safety Index for 150 Countries around the World

### 4.1. Evaluation of the COVID-19 Safety Index for 150 Countries

In this section, we first used the COVID-19 infection data from January to April 2022, together with the health resource carrying capacity of each country, to generate an evaluation of the COVID-19 safety index based on the epidemic situation in the first four months of 2022. Then, to further validate the reliability of the COVID-19 safety index for each country, the authors used the actual COVID-19 infections in each country from April to August 2022 to correlate the COVID-19 safety index scores based on the infections in the first four months of 2022 to justify the index. Finally, a machine learning model was used to predict the number of COVID-19 infections in the next year and to calculate the safety index for each country based on the predicted epidemic data, with a view to making some modest contribution to the prevention of epidemics in countries around the world in the future. 

#### 4.1.1. Evaluation of HRCC Indicators in 150 Countries

This paper evaluates the HRCC scores of 150 countries around the world and provides descriptive statistics on each of the HRCC evaluation indicators for the 150 countries (Table 6) in order to further analyze the regional differences that exist in each indicator.

Table 3 shows that the current gap between rich and poor is the most significant in the world, with the most valuable ratios of fiscal expenditure and income reaching as much as fifty thousand and sixty thousand times greater, respectively. In addition, the COVID-19 vaccination rate and population density are two indicators that are second only to government financial indicators in terms of their large differences, reaching more than two thousand and three thousand times higher, respectively. 

**Figure 9:**
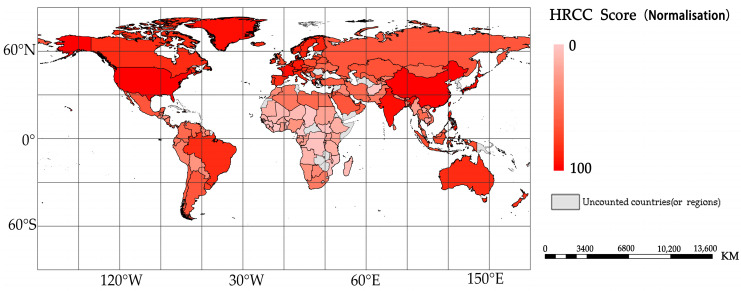
HRCC scores for 150 countries.

In this paper, the carrying capacity of healthcare resources for each country is presented in a map in Figure 9. The darker the color, the higher the HRCC score of the country. The map shows that the carrying capacity of healthcare resources is generally high in eastern Asia, northern Europe, and most regions of North America and Australia. In contrast, the carrying capacity of healthcare resources is relatively low in regions such as Africa, South America, and South East Asia, with African countries generally having low carrying capacity for healthcare resources. 

#### 4.1.2. Evaluation of COVID-19 Safety Index Scores in 150 Countries

The global emergence and rapid spread of Omicron worldwide, which began in late 2021 and early 2022, put each country at significant risk of infection and under pressure to prevent and control the epidemic in early 2022.

Therefore, after obtaining the HRCC scores for each country, we calculated the infection rate from January to April 2022 (the data from April to August are retained below to justify the indicator) to obtain the COVID-19 safety index score for each country. Due to the availability of epidemic data for each country, only the 139 countries with good availability were calculated. The top 15 countries in terms of COVID-19 safety index scores are shown Table 7.

The comparison of the indicators reveals that the COVID-19 safety index not only depends on the level of infection, but that the carrying capacity of medical resources also affects the safety of a region in responding to public health emergencies.

### 4.2. The Reasoned Argument Analysis of International Indicators of the COVID-19 Safety Index

After calculating the epidemic safety index scores for 150 countries from January to April 2022, we further assessed the COVID-19 safety index using least squares estimation (OLS). Precisely, the COVID-19 safety index was correlated with the actual status of the epidemic (four indicators were chosen to measure the infection status of the epidemic) in each country from April to August 2022, and the regression results are shown in Table 8.

The analysis in Table 8 shows a strong negative correlation between the epidemic safety index and the numbers of new confirmed diagnoses, cumulative deaths, and cumulative confirmed diagnoses; that is, the higher the level of safety, the lower the numbers of confirmed diagnoses and deaths. This suggests that the COVID-19 safety index is a good measure of a region’s risk level in responding to an epidemic and can provide a more accurate prediction of the extent of the epidemic over the next period. Thus, the validity of the ESI is again confirmed.

### 4.3. Predicting the COVID-19 Safety Index over the Next Year

**Figure 10:**
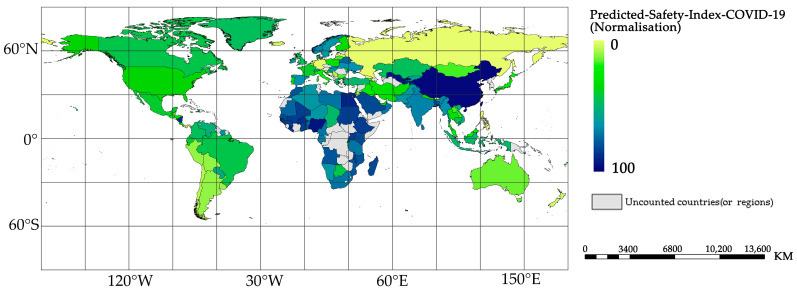
Predicted COVID-19 safety index scores of 150 countries.

Following the justification of the COVID-19 safety index, this paper presents a prediction of the outbreak safety index scores for countries worldwide in the coming year (macroscopic prediction and identification of vulnerabilities in response to recent outbreaks of Omicron variants on various continents). The results are presented in Figure 10.

The predictions show that some countries in Africa and Asia have high security index scores; as in North America, most countries in Northern Europe are more in the median, while some countries in South America, Australia, and Eastern Europe have low ratings. Combined with Figure 9, it can be seen that except for Africa, most countries in Asia, Europe, and the Americas have a close correlation between their epidemic safety index and their HRCCs, i.e., countries with higher HRCCs tend to be safer. It is important to note that the high COVID-19 safety index score for Africa is due to its low predicted infection rate. Therefore, Africa is not “actually safe” and is at significant risk of epidemic importation and infection. This also reminds us that the analysis of the epidemic safety index should be performed in the context of local epidemic control policies and the actual infection situation.

## 5. Discussion

As the research continues, the studies addressing COVID-19 risk assessments have become extensive and in-depth. Most of these studies have conducted risk assessments with human subjects, with studies on simulations of crowd behavior [22], evaluations of risk based on population mobility patterns using big data [23], research on mass risk resistance at the psychological level [24], and risk evaluations for vulnerable areas such as ports [26]. However, from a regional macroscopic perspective, there is a desperate need, particularly for developing countries, to build an epidemic risk evaluation system relying on the key resources of urban medical resources for epidemic prevention and control.

Meanwhile, unlike previous studies that focused on urban–rural differences [31], population differences [69], and differences in specific resource types [70], this paper focuses on the overall performance in terms of regional healthcare, financial, and demographic resilience aspects. Based on systematic empirical data and a model analysis, this study obtained the evaluation of the carrying capacity of healthcare resources and the scores of the epidemic safety index for each province and city in China and each country around the world at different stages of the epidemic, respectively. At the same time, the applicability of our constructed epidemic risk evaluation system was verified at all stages of the epidemic.

This study focused on the whole process of COVID-19 since the emergence of the outbreak, whilst two representative outbreak development stages were selected for the validation of the index. In this project, an epidemic safety evaluation index based on the medical resources carrying capacity and the prediction of the infection rate during the period was obtained. The proposed index melds the actual situation of the epidemic and the deep learning model, which provides a rapid and accessible epidemic safety evaluation index in response to public health emergencies in areas where outbreaks have occurred or are being exposed to relevant challenges. It contributes to identifying the weak areas of epidemic control and enhances the understanding of particular weaknesses, assisting in the further development of epidemic prevention measures. A specific discussion of each result is presented below.

### 5.1. Discussion of the Results for China

#### 5.1.1. Issues Revealed by the Study Results

(1) HRCC

An analysis of the HRCC scores and the evaluation results of each indicator for each province and city in mainland China revealed the following issues.

Firstly, there is a clear imbalance in the distribution of resources between provincial administrative regions, with differences being more pronounced at the provincial administrative level. For example, although Guangzhou has a good overall resource carrying capacity, the resources are mainly concentrated in Guangzhou and Shenzhen, among others. Secondly, economically developed provincial capitals or subprovincial cities are not necessarily the best human resources centers. This reminds us that economically strong cities may also face pressure on their resource carrying capacity due to the large populations they serve. Thirdly, areas with relatively high doctor-to-nurse ratios do not necessarily mean that the health workforce is well structured. The underlying reason behind this may also be an insufficient number of doctors, as in Xinjiang and most parts of Tibet.

(2) COVID-19 Safety Index

An analysis of the COVID-19 safety index evaluation for the first round of the 2020 epidemic revealed the following factors.

First, due to the unpredictable and widespread nature of the epidemic, there is a high probability of a “black swan” during the transmission phase, leading to the wide spread of the epidemic in a given area. The results of the epidemic safety index, which considers the impacts of epidemic factors, show differences in resilience between provinces and municipalities. Secondly, the impact of the epidemic risk should not be ignored in areas with higher scores, such as in Tibet and Qinghai, where the safety index is relatively high due to the smaller scale of actual infections. It is worth being aware that the safety index scores of the epidemic prevention system vary considerably within the provinces. Finally, when combined with the HRCC scores, this is further evidence that the real threat to the security of regional health systems does not depend solely on the scale of infectious diseases or the risk of epidemics. The adequacy, distribution, and allocation of key health resources and corresponding economic resources are also at fault.

#### 5.1.2. Contribution of the Findings in This Section

After thoroughly discussing the results of the HRCC and COVID-19 safety index scores for each province and city in mainland China, the following contributions were identified.

The HRCC index allows for a material-level evaluation of the abundance, development potential, potential risk, and equity of distribution of urban healthcare resources across and within provinces in China, allowing for the targeted identification of weaknesses in the healthcare resource system of each region in the fight against epidemic shocks.

The results of the COVID-19 safety index evaluation can adequately reflect the specific causes of the outbreak in each region of China in the first round of the new coronary pneumonia epidemic in 2020 (proximity to infected areas or deficiencies in medical resources).

The results of the two indicators reveal the weaknesses of epidemic prevention in mainland China, which will provide a strong basis for formulating epidemic prevention policies and deploying medical resources in the next round of outbreaks caused by the Omicron variant.

### 5.2. Discussion of the Results for Countries around the World

#### 5.2.1. Issues Revealed by the Study Results

(1) HRCC

The HRCC evaluation scores and the distribution of indicators worldwide revealed the following factors.

Firstly, there are significant differences in the financial and demographic distributions of countries around the world when responding to public health emergencies. There is considerable variation in the ability to mobilize medical and household goods in response to public health emergencies and the risks faced in the face of new emergencies. Secondly, differences in vaccination rates for the new crown also expose countries with lower vaccination rates to significant risks of infection in the event of an outbreak of the Omicron variant.

(2) COVID-19 Safety Index

The COVID-19 safety index evaluation and prediction scores for countries around the world reflect the following issues.

Firstly, most countries in the world are still at significant risk of a new coronary pneumonia outbreak; secondly, similar to the situation in the western and northeastern regions of mainland China, the African region generally scores higher at the outbreak level, despite the absence of superior medical resources. This suggests that when analyzing regional outbreak safety, it is important to compare countries with similar outbreak levels and outbreak control policies (inconsistencies in current outbreak policies lead to significant differences in the transmission scale). ‘Blind spots’ with low HRCC scores but high COVID-19 safety index scores should be the focus, emphasizing the strengthening of healthcare systems in these regions, with vigilance against “black swan” outbreaks. Finally, the predicted scores for countries worldwide adequately reflect the current areas of weakness in epidemic prevention and control.

#### 5.2.2. Contribution of Research Findings

After thoroughly discussing the results of the HRCC and COVID-19 safety index evaluations in 150 countries worldwide, the following contributions were identified.

By evaluating the HRCC in 150 countries worldwide, the current uneven distribution of healthcare resources in the world was identified. The weakness of healthcare resources in each country in the face of the Omicron variant of the epidemic was pinpointed, providing a set of evaluation indicators and ideas about the resilience of health resources when faced with another worldwide public health emergency in the future.

Combined with the HRCC scores, it is possible to identify the difficulty of fighting the epidemic in each country in the current response to the new outbreak caused by the Omicron variant of the virus, as well as the specific causes of the current situation of the epidemic (base of infection, medical resources, regional mobility, etc.). This will help provide direction for the next steps in developing epidemic prevention policies.

The results of the COVID-19 safety index can be used as a basis for the international community to provide pre-emptive assistance and support to the most affected areas in the context of the current globalization.

#### 5.2.3. Discussion of the Experience of Epidemic Prevention and Control

(1) Limitations of the data

It is important to emphasize that the current data on epidemic infections in various countries are subject to differences in statistical caliber and underreporting and underinsurance in some countries [71]. This led to distortion of the results of the calculations of infection rates in some countries in January–April 2022 in this paper. This problem affected the quality of the data in this paper and the results of the evaluation of the epidemic risk in each country. The control of data quality should be strengthened in future research work.

(2) Lessons for future outbreaks and epidemics

In calculating the outbreak safety indicators for each country, the top 15 countries were listed in this paper. The list reflects, to a certain extent, the current risk level of each country in dealing with the epidemic. However, it must be noted that there are quite a few countries on the list due to their short infection periods (which have some distorted data). There are also some countries that despite having longer infection periods than other countries, have shown higher risk scores with their better HRCC scores. China, the top-ranked country, has an epidemic prevention policy worth studying. Until 8 January 2023, China had firmly implemented a dynamic clearance policy [72], which greatly controlled the infection rate. Additionally, countries such as China [73] and India [74] have firmly promoted vaccination. This action has also contributed to the distribution of medical resources and the control of infection rates. In addition, the epidemic safety index in Haiti is much higher than expected [75]. This may be because the Haitian population is immune due to the impact of the initial spread of the epidemic, making a larger percentage of the population immune [75]. It could also be the reason for the better vaccination rate in Haiti [76]. In summary, we found that the physical control of the epidemic, vaccination rates, and adequate medical supplies are the most important measures to prevent and control the current epidemic.

### 5.3. Discussion of Research Contributions

In summary, the contributions of this paper are as follows. Firstly, it provides a set of indicators to measure the carrying capacity of regional medical resources and the degree of risk of epidemics in the face of public health emergencies, contributing a new research perspective and direction to the study of risk identification and emergency management in public health. Secondly, the study of the first round of the 2020 epidemic in mainland China identified the weaknesses in the resistance to the epidemic in Chinese provinces and cities. It also provides strong recommendations and supporting evidence for formulating epidemic prevention policies and resource mobilization in China in response to the new virus variant. Thirdly, we identified and judged the current epidemic resistance dilemmas and epidemic risk levels faced by countries worldwide from a global perspective, providing strong direction and evidence for the international community to provide relevant relief. Finally, a prediction model was used to forecast the level of epidemic risk in countries around the world in the next six months, providing a focus and direction for the future work of national epidemic prevention policies.

## 6. Conclusions

Since the 21st century, public health emergencies such as SARS, H1N1, MARS, Ebola, and COVID-19 have been breaking out in human society [77], and the importance of research for public health emergencies has gradually become greater. In preventing and controlling such public health emergencies, the first thing to do is to evaluate and identify the degree of risk and the weaknesses of the epidemic resistance in different regions [8,9].

In this paper, based on regional medical resources, we constructed a set of evaluation systems for the carrying capacity of medical resources and the degree of risk in the face of regional public health emergencies. We took COVID-19 as an example and selected its two development stages with different characteristics as evaluation objects to evaluate the HRCC and epidemic risk degree for China and the world, respectively. At the same time, we selected the epidemic prevention and control performance of different epidemic stages, and the regression results verified the credibility of the index system. Based on the above, we predicted and evaluated the epidemic safety index in the next six months for each country around the world and provided a basis for the formulation of COVID-19 epidemic prevention and control policies in each country within a certain period in the future.

In addition, this paper proposes a research system and paradigm that combines medical resources and the infection rate during the epidemic in each place to demonstrate the degree of regional risk comprehensively. When more dimensions of healthcare resources and more accurate period infection rates are obtained, the system will be more accurate in evaluating the risk of regional outbreaks. The methodology and findings of this study provide a benchmark for future studies that evaluate outbreak risk by combining healthcare resources. It also has the potential to be applied widely, not only to the various phases of the COVID-19 outbreak but also to different public health emergencies.

However, the current study has several areas for improvement. First, in constructing the HRCC evaluation index system, the first stage of the three-level index construction for China and the second stage for international countries were selected for different indicators. This was partly due to the difference in statistical caliber between China and international countries, and partly since the research on the COVID-19 epidemic continues to deepen. The form of the epidemic continues to change, and resources such as the COVID-19 vaccines are gradually replacing the initial medical supplies as the most important forces in the fight against the epidemic. Second, the statistical calculation of infection rates during the epidemic in various countries worldwide should have considered the impact of data variation due to the inconsistency of specific local epidemic prevention and control policies. Future experiments will also focus on strengthening the quantitative study of epidemic prevention and control policies. Finally, with the continuous progress of technology and the development of the epidemic, the index system constructed in this paper needs to be updated according to the actual situation to adapt to new scenarios. In summary, a multiple-risk evaluation system based on the carrying capacity of medical resources will provide a more reliable basis for the prevention and control of public health emergencies in the future.

## Figures and Tables

**Table 1:** The indicator system of the HRCC model.

First Indicators	Secondary Indicators	Tertiary Indicators	
		China	International
Objective indicators of regional healthcare resources	Medical workforce resources	Medical technicians (total; per 10,000 population)	Medical technicians (total; per 10,000 population)
		Nursing and midwifery personnel (total; per 10,000 population)	Nursing and midwifery personnel (total; per 10,000 population)
		Medical doctors (total; per 10,000 population)	Medical doctors (total; per 10,000 population)
		CDC staff (total; per 10,000 population)	
	Medical equipment	Ventilators (total; per 10,000 population)	HAQ(Healthcare Access and Quality) Index
		CT (total; per 10,000 population)	
		Ambulance (total; per 10,000 population)	
	Medical facilities	ICU (total; per 10,000 population)	
		Hospital Beds (total; per 10,000 population)	
	Indicators of epidemic-specific medical resources	COVID-19 case intensive care hospital (total; per 10,000 population)	Vaccinations (total; per 10,000 population)
		Fever clinics (total; per 10,000 population)	
		Hospitals able to treat COVID-19 patients (total; per 10,000 population)	Average of 13 IHR (International Health Regulation) core capacity scores
	Financial indicators	Domestic general government health expenditure (GGHE-D) and it as percentage of general government expenditure (GGE) (total;%)	Domestic general government health expenditure (GGHE-D) and as A percentage of general government expenditure (GGE) (total;%)
		General government receipts (GGR) (total; per person/¥)	General government receipts (GGE) (total; per person/$)
		General government expenditure (GGE) (total; per person/¥)	General government expenditure (GGE) (total; per person/$)
Basic indicators of the regional population carrying capacity	Population indicators	Population density (total; per KM2)	Population density (total; per KM2)
		Aging rate (total; %)	Aging rate (total; %)

**Table 2:** Weight distribution of HRCC indicators in 31 provinces in mainland China.

Indicators	Weighting Results
Medical technicians	0.03050
Nursing and midwifery personnel	0.03296
Medical doctors	0.03894
CDC staff	0.05793
Ventilators	0.06623
CT	0.07420
Ambulance	0.07858
ICU	0.05110
Hospital beds	0.04119
COVID-19 case intensive care hospital	0.08111
Fever outpatient clinics(per 10,000 population)	0.08324
Hospitals able to treat COVID-19 patients	0.08111
General government health expenditure (GGHE-D) and it as percentage of general government expenditure (GGE)	0.04800
General government receipts (GGR)	0.10040
General government expenditure (GGE)	0.08154
Population density	0.01380
Ageing rate	0.03919

**Table 3:** Weight distribution of HRCC indicators in 150 countries.

Indicators	Weighting Results
Medical technicians	0.10592
Nursing and midwifery personnel	0.10957
Medical doctors(per 10,000 population)	0.13880
HAQ (Healthcare Access and Quality) Index	0.02146
Vaccinations	0.19891
Average of 13 International Health Regulations core capacity scores	0.00892
Domestic general government health expenditure (GGHE-D) and it as percentage of general government expenditure (GGE)	0.11732
General government receipts (GGE)	0.14966
General government expenditure (GGE)	0.14421
Population density (per KM2)	0.00104
Ageing rate (percentage of people over 65 years old)	0.00420

**Table 4:** Descriptive statistics of HRCC indicators for 31 provinces in mainland China.

Variables	Observed Values	Average Values	Standard Deviation	Maximum	Minimum	Ratio of Maximum and Minimum Values
Density of Medical technicians (per 10,000 population)	31	68.022	14.085	116.900	22.812	5.125
Density of nursing and midwifery personnel (per 10,000 population)	31	29.450	5.190	49.034	15.276	3.210
Density of medical doctors (per 10,000 population)	31	25.964	5.231	45.588	15.276	2.984
Density of CDC staff (per 10,000 population)	31	1.581	0.564	3.651	0.841	4.343
Density of Ventilators (per 10,000 population)	31	0.263	0.215	1.407	0.672	2.095
Density of CT (per 10,000 population)	31	0.964	0.197	1.176	0.101	11.598
Density of Ambulance (per 10,000 population)	31	0.205	0.079	0.455	0.131	3.461
Density of ICU Beds (per 10,000 population)	31	0.418	0.115	0.760	0.197	3.852
Density of Hospital Beds (per 10,000 population)	31	59.770	8.869	73.818	41.020	1.800
Density of COVID-19 case intensive care hospital (per 10,000 population)	31	0.015	0.012	0.041	0.00042	97.170
Density of fever outpatient clinics (per 10,000 population)	31	0.085	0.075	0.313	0.00678	46.213
Density of hospitals able to treat COVID-19 patients (per 10,000 population)	31	0.090	0.073	0.313	0.00958	32.734
Domestic general government health expenditure (GGHE-D) as a percentage of general government expenditure (GGE) (%)	31	0.015	0.114	0.064	1.771	0.091
general government receipts (GGR)	31	5541.326	28,330.545	3426.650	8.268	7713.414
general government expenditure (GGE)	31	9195.249	54,019.353	9276.588	5.823	16,829.923
Population density(per KM2)	31	467.498	711.597	3947.761	2.971	1328.870
Ageing rate(Percentage of people over 65 years old)(%)	31	0.130	0.029	0.174	0.057	3.072

Data sources: China Health Statistical Yearbook; Provincial Municipal Health Committee Bulletins; Provincial “National Economic and Social Development Statistical Bulletins”; China’s Seventh Population Census Bulletin.

**Table 5:** Correlation analysis between the COVID-19 safety index and the actual performance index scores of the epidemic in 333 cities.

	(1)	(2)	(3)	(4)	(6)	(7)
	Time to Peak Infection	Cumulative Number of Infections	Duration of the Outbreak	Peak Number of Infections	Number of Deaths	Full Recovery Time
COVID-19 safety index	−0.000 *** (−3.93)	−0.018 (−1.40)	−0.001 *** (−6.31)	−0.004 (−1.20)	−0.001 (−1.14)	−0.001 *** (−7.55)
_cons	11.177 *** (15.86)	407.545 (1.51)	28.870 *** (20.92)	91.244 (1.27)	24.266 (1.17)	46.595 *** (50.51)
R2	0.032	0.004	0.103	0.003	0.003	0.149
N	302	302	302	302	302	302

Note: The results in the above table are regressed based on robust labeling errors, with *t*-values in parentheses and significance levels of *** *p* < 0.01.

**Table 6:** Descriptive statistics of HRCC indicators for 150 countries worldwide.

Variables	Average Values	Standard Deviation	Maximum	Minimum	Ratio of Maximum and Minimum Values
Density of Medical technicians (per 10,000 population)	4.357	4.527	19.800	0.100	198.000
Density of nursing and midwifery personnel (per 10,000 population)	48.176	48.409	229.500	2.000	114.750
Density of medical doctors (per 10,000 population)	19.634	17.118	70.900	0.300	236.333
HAQ(Healthcare Access and Quality) Index	60.607	22.692	97	23	4.217
Vaccinations rate (per 10,000 population)	7725.635	8805.657	33,493.151	9.267	3614.072
Average of 13 International Health Regulations core capacity scores	65.487	18.200	100.000	20.000	5.000
Domestic general government health expenditure (GGHE-D) as percentage of general government expenditure (GGE) (%)	10.510	5.128	24.200	0.600	40.333
general government receipts (GGR)	4257.978	7494.248	41,293.93	0.655	63,052.933
general government expenditure (GGE)	4578.553	7507.530	40,766.773	0.707	57,693.270
Population density (per KM2)	211.130	709.377	8158.996	2.096	3893.095
Ageing rate (Percentage of people over 65 years old) (%)	940.667	686.838	2900.000	100	29

Data source: World Health Statistics 2022, Oxford University’s “Our World in Data” database.

**Table 7:** Top 15 countries in the COVID-19 safety index.

Continent	Country	HRCC	Period Infection Rates	COVID-19 Safety Index (Normalization)
Asia	China	0.528	0.0000741	100
Asia	Tajikistan	0.036	0.0000307	16.604
Africa	Nigeria	0.031	0.0000689	6.335
Central America	Nicaragua	0.041	0.0001255	4.658
Africa	Niger	0.017	0.0000659	3.618
Africa	United Republic of Tanzania	0.019	0.0000769	3.559
Africa	Sierra Leone	0.016	0.0000875	2.691
North America	Haiti	0.072	0.0004126	2.467
Africa	Democratic Republic of the Congo (DRC)	0.021	0.0001365	2.209
Africa	Chad	0.014	0.0001041	1.916
Africa	Burkina Faso	0.018	0.0001556	1.707
Asia	East Timor	0.266	0.0022989	1.628
Africa	Benin	0.014	0.0001287	1.610
Africa	South Sudan	0.015	0.0001649	1.327
Africa	Liberia	0.021	0.0002285	1.278
Asia	India	0.374	0.0059688	0.879

**Table 8:** Correlation analysis between the COVID-19 safety index and the actual performance index of the epidemic in 150 countries.

	(1)	(2)	(3)	(4)
	Number of New Deaths	Number of New Confirmed	Cumulative Number of Deaths	Cumulative Number of Confirmed
COVID-19 safety index	−0.003 (−1.398)	−0.904 ** (−2.547)	−7.863 *** (−4.872)	−677.516 *** (−5.232)
_cons	14.984 *** (12.226)	5172.349 *** (23.351)	45,520.358 *** (45.201)	3,940,000 *** (48.783)
R2	0.000	0.000	0.002	0.002
N	15,599	15,599	15,599	15,599

Note: The results in the above table are regressed based on robust labeling errors, with *t*-values in parentheses and significance levels of *** *p* < 0.01, ** *p* < 0.05.

## Data Availability

Not applicable.

## Appendix A

**Table A1:** A correlation analysis of COVID-19 safety index (calculated separately for each weight assignment method) and outbreak control performance scores.

	Cumulative Number of Infections	Duration of the Outbreak	Peak Number of Infections	Number of Deaths	Time to Peak Infection	Full Recovery Time
COVID-19 safety index (Entropy-weight TOPSIS method)	−0.034	−0.279 ***	−0.032	−0.027	−0.182 ***	−0.335 ***
COVID-19 safety index (Analytic Hierarchy Process)	−0.022	−0.153 ***	−0.021	−0.018	−0.099 *	−0.201 ***
COVID-19 safety index (Factor analysis method)	−0.021	−0.149 ***	−0.02	−0.017	−0.097 *	−0.196 ***
COVID-19 safety index (the Grey Relational Analysis)	−0.032	−0.169 ***	−0.026	−0.021	−0.129 **	−0.272 ***

Note: The results in the table above are based on a correlation analysis of Pearson’s coefficient, with *t*-values in parentheses and significance levels of *** *p* < 0.01, ** *p* < 0.05, and * *p* < 0.1.
